# First georeferenced record of *Spinolambrus macrochelos* (Herbst, 1790) (Arthropoda, Parthenopidae) from the type locality (Genoa, Italy), with reference to the neotype designation

**DOI:** 10.3897/BDJ.14.e174523

**Published:** 2026-03-04

**Authors:** Alice Guzzi, Giovanni Roppo Valente, Federico Vignati, Stefano Schiaparelli

**Affiliations:** 1 Department of Earth, Environmental and Life Sciences (DISTAV), University of Genoa, Corso Europa 26, Genoa, Italy Department of Earth, Environmental and Life Sciences (DISTAV), University of Genoa, Corso Europa 26 Genoa Italy; 2 National Biodiversity Future Center (NBFC), Palermo, Italy National Biodiversity Future Center (NBFC) Palermo Italy; 3 Italian National Antarctic Museum (MNA, Section of Genoa), University of Genoa, Viale Benedetto XV No. 5, Genoa, Italy Italian National Antarctic Museum (MNA, Section of Genoa), University of Genoa, Viale Benedetto XV No. 5 Genoa Italy

**Keywords:** Mediterranean Sea, occurrence, open data, museum collections

## Abstract

*Spinolambrus macrochelos* (Herbst, 1790) is a Mediterranean parthenopid crab whose original type material has been lost. A male specimen from Genoa (Italy) was designated as neotype by Tan & Low (2014), establishing the species’ type locality. Despite this taxonomic clarification, no georeferenced records from the type locality are currently available in global biodiversity aggregators (GBIF, OBIS). We report a new occurrence of *S. macrochelos* from the same area as the neotype, providing morphological documentation, georeferenced metadata and openly available occurrence data. This constitutes the first publicly accessible record for the species from its type locality.

## Introduction

The crab *Cancer macrochelos* Herbst, 1790 (type species by original designation) was first described from the Mediterranean Region near Naples (“im neapolitanischem Meere”) (Tan and Ng 2007), but the original type material has since been lost (Sakai 1999). In order to stabilise the species’ taxonomy, Tan and Low (2014) selected a male specimen from the waters off Genoa, Italy (RMNH D 43604) as the neotype. This action also established *Lambrus spinosissimus* Osório, 1923 as an objective synonym of *Spinolambrus macrochelos* in accordance with the International Code of Zoological Nomenclature. The designation fixed the type locality of *Spinolambrus macrochelos* (Herbst, 1790) as the Mediterranean Sea off Genoa. This species is known from the Mediterranean Sea and parts of the Eastern Atlantic, with records ranging from the Portuguese coast southwards to Morocco (Tan 2004, Tan and Ng 2007). It inhabits sandy or muddy substrates from very shallow waters to depths exceeding 700 m, although most occurrences are between 18 and 370 m (Holthuis and Gottlieb 1958, Guerao and Abelló 1999, Politou et al. 2003, Fanelli et al. 2007, Hasan et al. 2008). Reports from the eastern basin include localities in the Aegean Sea, Levantine coasts, Cyprus and Turkiye (Koukouras et al. 1992, Fishelson 2000, Kocatas et al. 2001, Kocatas and Katagan 2004, Özcan and Katağan 2009, AteŞ et al. 2010). Although the range is relatively broad, the species is considered uncommon (Falciai and Minervini 1992). Despite the high relevance of the Genoa locality for this species, there are currently no georeferenced records from this area available through global biodiversity infrastructures, such as GBIF or OBIS. This absence restricts the accessibility of verified reference material for taxonomic confirmation, distributional assessments and ecological analyses. In this work, we report a new occurrence of *S. macrochelos* from the vicinity of the neotype locality. The record includes precise geographic coordinates, detailed morphological documentation, the creation of a digital twin of immediate availability for morphological comparisons and the inclusion of the physical specimen in a curated collection. The data are formatted according to Darwin Core standards and are openly accessible through biodiversity aggregators, in accordance to FAIR principles (Findable, Accessible, Interoperable and Reusable).

## Materials and Methods

**Figure 1.**
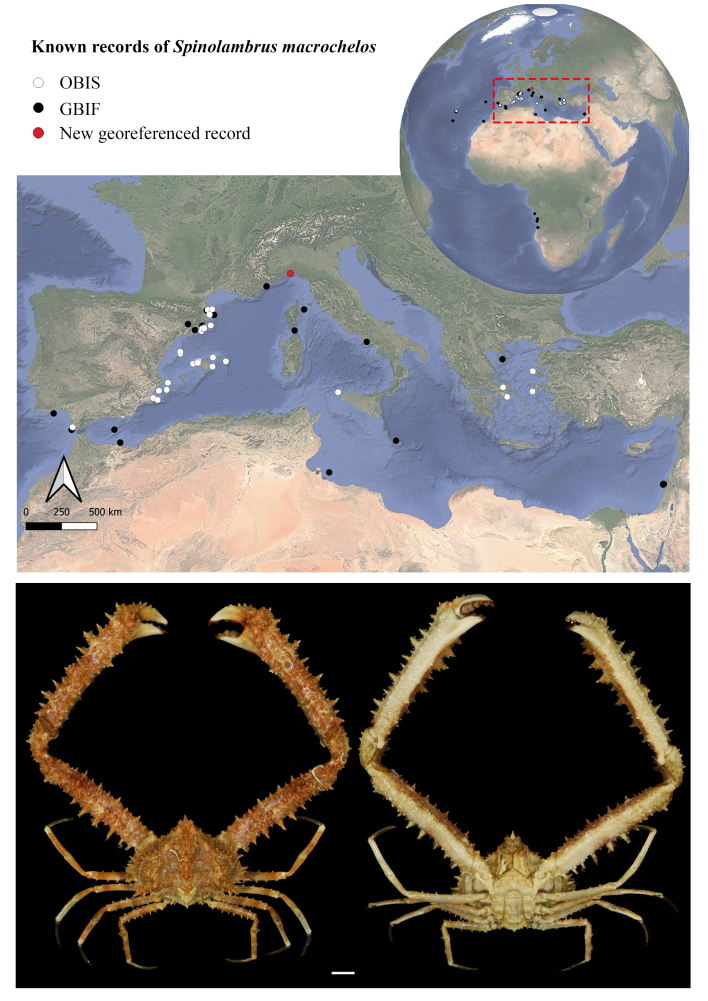
Available georeferenced distributional data of the species were obtained from global open-access data repository: OBIS www.obis.org (in white) and GBIF www.gbif.org (in black). *Spinolambrus macrochelos* (Herbst, 1790) IZUG-16144 collection site (red point) in the Ligurian Sea (Mediterranean Sea). Specimen dorsal (left) and ventral (right) view. Scale 1 cm.

**Figure 2.**
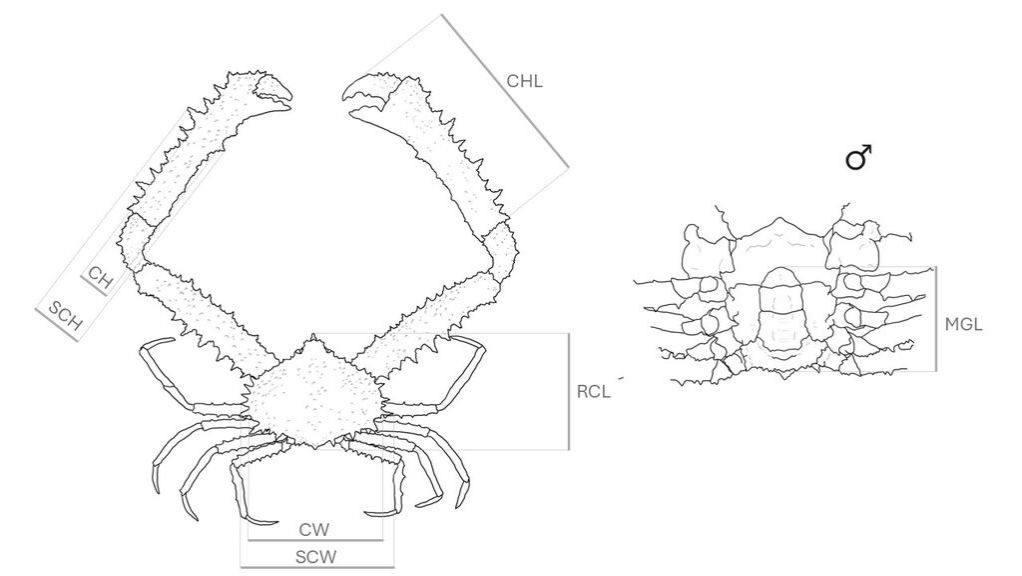
A schematic of morphometric measurements. Left dorsal view: rostral carapace length (RCL), carapace width (CW), spinal carapace width (SCW), claw length (CHL), claw height (CH) and spinal claw height (SCH). Right ventral view (male): male gonopod length (MGL).

A male specimen of *S. macrochelos* was collected by one of the authors (F.V.) on 10 July 2025, off Genoa (Lat 44°20'39.9"N, Long 8°53'29.4"E, Fig. 1) during a trammel net survey conducted at an average depth of 116 m. After capture, the specimen was transferred to the laboratory, where diagnostic morphological traits of the organism were documented through high-resolution photography in accordance with national protocols established under the National Recovery and Resilience Plan (PNRR) framework (Guzzi and Schiaparelli 2024). The specimen was identified to the lowest possible taxonomic resolution, based on morphology, using available literature and an identification key (Falciai and Minervini 1992, Tan and Ng 2007). It was distinguished from *Spinolambrus notoalis* (Manning & Holthuis, 1981) by the presence of distinctly narrow, triangular spines along the outer margin of the manus, each bearing numerous smaller subsidiary spines. Prior to preservation, biometric measurements were taken using dial calipers (precision ± 0.1 mm). The parameters recorded (Fig. 2) comprise the following: rostral carapace length (RCL), dorsally along the mid-line from the tip of the rostrum to the posterior carapace margin; carapace width (CW); spinal carapace width (SCW), across the widest part of the carapace; male gonopod length (MGL), with the abdomen fully opened, along the entire gonopod as it was held against the sternites, from its junction at the expanded base to the spiny, curved tip, distally; claw length (CHL); claw height (CH) and spinal claw height (SCH). Furthermore, the preservation of a tissue sample under standardised protocols ensures that the specimen will also serve as a genetic reference for future molecular investigations. A tissue subsample was collected for future molecular analyses, following standardised procedures also developed under the PNRR initiative (Guzzi and Schiaparelli 2024), ensuring methodological uniformity and enabling future integration of genetic data. The specimen was then prepared for long-term preservation and display through taxidermic techniques. This process included removal of internal soft tissues, insertion of a wire-based support structure, application of internal padding and final mounting to maintain the original three-dimensional morphology. Following preparation, the specimen was accessioned into the Zoological Collections of the University of Genoa, curated by the Department of Earth, Environmental and Life Sciences (DISTAV), under voucher number IZUG-16144. The occurrence reported in this article is deposited at GBIF, Global Biodiversity Information Facility: https://doi.org/10.15468/znabpn. For the 3D digital twin generation, a comprehensive series of overlapping photographs was acquired from multiple perspectives using the Orbitvu Alphashot 360 system. Images were taken with a Canon EOS R camera, equipped with an RF 24–105 mm lens. All acquisitions were made under diffuse lighting conditions to ensure homogeneous illumination and accurate colour reproduction. The photographic dataset was then imported in Agisoft Metashape v.1.8.3, for photogrammetric reconstruction. The final 3D model is available in the Sketchfab collection of 3D models from the University Museum System of the Genoa University at the following link: https://skfb.ly/pBxn9. Available georeferenced distributional data of the species were obtained from global open-access data repository (OBIS www.obis.org and GBIF www.gbif.org) .

## Results and Discussion

The distribution of *Spinolambrus macrochelos* extends from the Atlantic Ocean, with records along the Angolan coast to the Mediterranean Sea (Fig. 1). Given the species' wide Mediterranean distribution, spanning from shallow to great depths, the specimen presented here, collected at 116 m, aligns with the established ecological range of *S. macrochelos* (Corsini-Foka and Pancucci-Papadopoulou 2012). A comprehensive examination of the presently available georeferenced distributional data for the species retrieved from global open-access biodiversity repositories reveals a substantial paucity of spatially explicit information. From GBIF, 137 occurrence records were identified, of which only 47 include valid georeferencing. OBIS contributes with 40 records, all of which are georeferenced. Notably, neither source provides any documented occurrences from the Ligurian Sea. The occurrence (Fig. 1) of this species in this area is reported in the "Checklist of the Italian Fauna" (Crocetta et al. 2021). However, also this record lacks supporting spatial detail, as no precise geographical coordinates or locality data are provided. While the citation substantiates the species’ presence within the broader Ligurian Basin, the absence of verifiable georeferenced information constrains efforts to delineate its exact distribution and to characterise its habitat range within the region. For completeness, a verification was also conducted within the zoological collection of the Giacomo Doria Natural History Museum in Genoa (accessed 2 October 2025), the principal institution responsible for preserving zoological specimens from this region of Italy. The absence of records for this species indicates a lack of historical material in the collection and suggests potential gaps in past sampling efforts within the Ligurian Sea. This further underscores the significance of the record presented here, which constitutes a verifiable, georeferenced occurrence from the same area of the type locality of *Spinolambrus macrochelos*. Given the proximity to the capture site of the designated neotype by Tan and Low (2014), this specimen provides a valuable reference point for comparative morphological analyses. All detailed measurements are summarised in Table 1, while its digital twin offers an additional resource for visual comparison and the assessment of potential intraspecific variation relative to the type material. By publishing the occurrence through global biodiversity infrastructures, such as GBIF and OBIS in Darwin Core-compliant format, this study addresses a critical gap in the species’ georeferenced distributional data. Locality-confirmed records from the type region of *S. macrochelos* are particularly important, as they provide authoritative benchmarks for taxonomic validation, facilitate accurate distribution mapping and strengthen the reliability of biodiversity assessments in the future. Finally, by coupling traditional specimen preservation with 3D digitisation and open-access data publication, this work exemplifies how modern curatorial practices can enhance the scientific, educational and conservation value of natural history collections, ensuring their relevance for future biodiversity research.

## Funding

This work was partially funded by the National Recovery and Resilience Plan (NRRP), Mission 4 Component 2 Investment 1.4—Call for tender No. 3138 of 16 December 2021, rectified by Decree No. 3175 of 18 December 2021 of the Italian Ministry of University and Research funded by the European Union—NextGenerationEU. Project code CN_00000033, Spoke 1, Concession Decree No. 1034 of 17 June 2022 adopted by the Italian Ministry of University and Research, Project title “National Biodiversity Future Center—NBFC” (G. Bavestrello).

## Figures and Tables

**Table 1.** Specimen morphometric measurements.

**Position**	**Section**	**Code**	**Length (cm)**
Dorsal	Rostral carapace length	RCL	4.1
	Carapace width	CW	1.8
	Spinal carapace width	SCW	5.4
	Claw length 1 (left)	CHL₁	7.1
	Claw height 1 (left)	CH₁	1.1
	Spinal claw height 1 (left)	SCH₁	1.9
	Claw length 2 (right)	CHL₂	7.3
	Claw height 2 (right)	CH₂	1.1
	Spinal claw height 2 (right)	SCH₂	1.8
Ventral	Male gonopod length	MGL	1.7
